# Supplementary material for: Premature skewing of T cell receptor clonality and delayed memory expansion in HIV-exposed infants
Source: Nat Commun. 2024 May 14;15:4080. doi: 10.1038/s41467-024-47955-5 (PMC11093981; doi:10.1038/s41467-024-47955-5)
Supplement: Supplementary file 1 — Supplementary Information [file 41467_2024_47955_MOESM1_ESM.pdf]

Supplementary information

Premature skewing of T cell receptor clonality and delayed memory expansion in HIV-exposed infants.

Sonwabile Dzanibe[1], Aaron J. Wilk[2], Susan Canny[2,3], Thanmayi Ranganath[2], Berenice Alinde[1,4], Florian Rubelt[5,6], Huang Huang[5,6], Mark M. Davis[5,6,7], Susan Holmes[8], Heather B. Jaspan[1,9**], Catherine A. Blish[2,6,10**] and Clive M. Gray[1,4**]

<u>Supplementary Tables</u>

- Supplementary Table S1 Demographic characteristic of HIV-uninfected and HIV-infected mothers and their respective HIV uninfected-unexposed infants (iHUU) and HIV-exposed uninfected infants (iHEU).
- Supplementary Table S2 List of key resource materials used in the study.
- Supplementary Data S1 Summary of T cell receptor sequencing quality control parameters used for naïve and memory T cells measured at birth, and weeks 4, 15 and 36. *p-value<0.05; two-tailed Wilcoxon test, adjusted for multiple comparisons using FDR.

Table S1 Demographic characteristic of HIV-uninfected and HIV-infected mothers and their respective HIV uninfected-unexposed infants (iHUU) and HIV-exposed uninfected infants (iHEU).

**Demographics**	**iHUU (N=16)**	**iHEU (N=40)**	**P***
**Mother**			
Median maternal age at delivery, years (range)	23.5 (19-39)	30.5 (19-39)	0.02
Median gestational age, weeks (range)	39.0 (36-41)	39.0 (36-42)	0.9
Median maternal CD4 count cells/μL (IQR)		418.0 (285.0-526.0)	
Median maternal viral load copies/mL (IQR)		20.0 (20.0-70.0)	
**Infant**			
Median birth weight, kg (IQR)	3.2 (2.8-3.5)	3.1 (2.9-3.4)	0.8
Sex, female (%)	8 (50.0)	14(35.0)	0.4
Median duration of breastfeeding (IQR)	44.3 (36.0-52.0)	52	0.1
Median duration of exclusive breastfeeding, weeks (IQR)	12.5 (9.3-27.0)	17.5 (6.3-36.0)	0.8
Completed rotavirus vaccination (%)	13 (81.3)	35 (87.5)	0.7
Completed pertussis vaccination (%)	12 (75.0)	33 (82.5)	0.7

* Two-tailed Wilcoxon test used to compared differences between iHUU and iHEU, except for comparing distribution by sex and vaccine completion status in which p-values was computed using χ^2 test.

Table S2 List of key resource materials

Mass cytometry antibodies				
Antigen	Metal isotope	Primary antibody source	Catalogue	Dilution
Extracellular antibodies				
CD19	In115Di	Biolegend	302247	1:20
CD20	In115Di	Biolegend	302343	1:20
CD14	Nd150Di	Biolegend	301843	1:5
CD3	Nd142Di	Biolegend	300443	1:10
CD4	Tb159Di	Biolegend	317402	1:80
CD8	Nd144Di	Biolegend	344727	1:40
CD45RA	Nd148Di	Biolegend	304143	1:40
KIR2DL1	Sm149Di	R&D Systems	328302	1:40
CD57	Eu151Di	Biolegend	359602	1:40
Siglec-7	Eu153Di	Biolegend	339202	1:20
PD-1	Sm154Di	Biolegend	329941	1:10
NKp46	Gd155Di	Biolegend	331902	1:20
NKG2D	Gd156Di	Biolegend	320802	1:5
NKG2C	Gd157Di	R&D System	MAB138	1:40
2B4	Gd158Di	Biolegend	329502	1:40
CXCR3	Gd160Di	Biolegend	353733	1:10
NKp30	Dy161Di	Biolegend	325202	1:10
CD39	Dy162Di	Biolegend	328221	1:40
KIR3DL1	Dy163Di	BD biosciences	555964	1:40
TIGIT	Dy164Di	R&D System	MAB7898	1:20
CD16	Ho165Di	Biolegend	302051	1:20
CD69	Er166Di	Biolegend	310939	1:20
CD127	Er167Di	Biolegend	351337	1:40
CCR7	Er168Di	Biolegend	353237	1:5
NKG2A	Tm169Di	Fluidigm	3169013B	1:20
KIR2DL3	Er170Di	R&D System	MAB2014	1:5
CCR4	Yb171Di	BD Biosciences	551121	1:5
NTBA	Yb172Di	Biolegend	317202	1:20
CCR6	Yb173Di	Biolegend	353427	1:20
CD56	Yb174Di	BD Biosciences	559043	1:20
CD25	Lu175Di	Biolegend	356102	1:20
CD38	Yb176Di	Biolegend	303535	1:20
CD7	La139Di	Biolegend	343111	1:20
DNAM1	Pr141Di	BD Biosciences	559787	1:5

LILRB1	Nd143Di	R&D System	MAB20172	1:5
CD27	Nd146Di	Biolegend	302839	1:40
HLA-DR	Cd112Di	Biolegend	361602	1:25
Intracelular antibodies				
Ki67	Sm152Di	Biolegend	350523	1:10
Perforin	Sm147Di	Abcam	ab47225	1:10
FcERIγ	Nd145Di	Millipore	06-727	1:5
Live/Dead and DNA markers				
Cisplatin	Pt	Fluidigm	201195	
DNA intercalator	Ir	Fluidigm	201192B	
**Fluorescent Activated Cell Sorting**				
Antigen	Fluorochrome	Source	Catalogue	
CD3	FITC	Biolegend	317306	1:50
CD4	Alexa Flour 700	Biolegend	344622	1:50
CD8	BV711	Biolegend	301043	1:20
CD45RA	PE-Texas red	Invitrogen	MHCD45RA17	1:20
CD27	PE-Cy5	Biolegend	302858	1:80
CCR7	PE-Cy7	Biolegend	353225	1:40
**T cell receptor RNA sequencing**				
		Source	Catalogue	
RNAProtect solution		Qiagen	76104	
RNAeasy Plus Micro Kit		Qiagen	74034	
SMARTScribe Reverse Transcriptase		Takara Bio	639538	
Q5 Hot Start Master Mix		NEB	M0494S	
Advantage 2 Polymerase		Takara Bio	639201	
Primers				
isoC-5′-GTCAGATGTGTATAAGAGACAGnnnnnnnnnnCGATAGrGrGrG-3′-C3_Spacer		IDT DNA	Custom order	
Poly A tail 5′- GTGTCACGTACAGAGTCATCttttttttttttttttttttttttttttt-3′ VN		IDT DNA	Custom Order	
**Antibody quantification**				
		Source	Catalogue	
Anti-Pertussis IgG ELISA Kit		Abcam	ab108709	
Anti-Rotavirus IgA rabbit antibodies		Abcam	ab93860	
Rotavirus strains RV3 and 8912		NIH		
MA104 cells		NIH		

biotyinylated goat anti-human IgA	Jackson Laboratories	09-065-011	
peroxidase conjugated avidin:biotin	Vector Laboratories	SP-3010-1	
O-phenylenediamine	Sigma	P8287-50TAB	
**General reagents**			
	Source	Catalogue	
Histopague Ficoll	ThermoFisher	17-1440-03	
Fetal Bovine Serum	Corning	MT35016CV	
Benzonase	EDM Millipore	70664	
EQ Four element calibration beads	Fluidigm	201078	
eBioscience permeabilization buffer	eBioscience	00-8333-56	
Paraformaldehyde	ThermoFisher	15710	

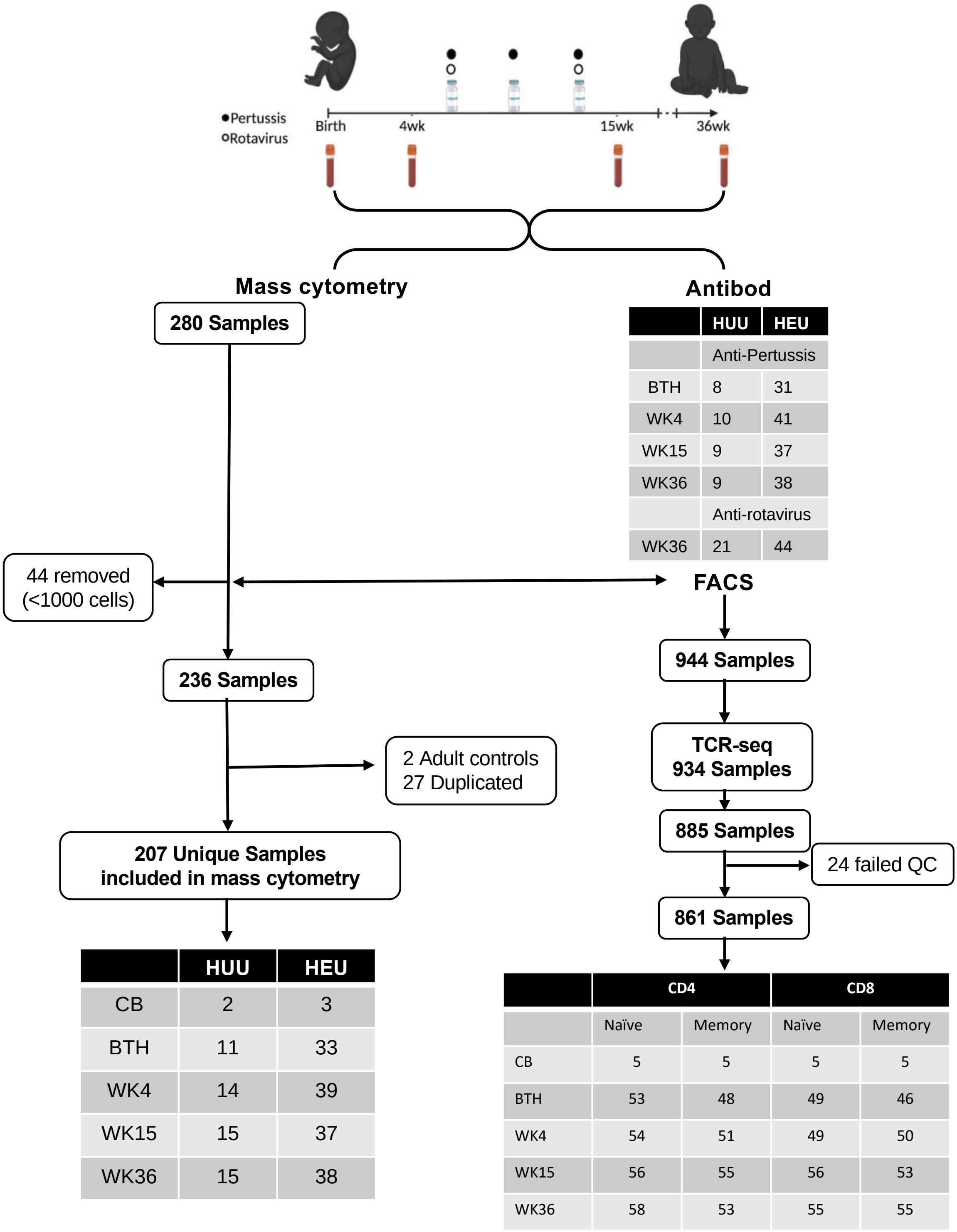

	HUU	HEU
	Anti-Pertussis	
BTH	8	31
WK4	10	41
WK15	9	37
WK36	9	38
	Anti-rotavirus	
WK36	21	44

	HUU	HEU
CB	2	3
BTH	11	33
WK4	14	39
WK15	15	37
WK36	15	38

	CD4		CD8	
	Naïve	Memory	Naïve	Memory
CB	5	5	5	5
BTH	53	48	49	46
WK4	54	51	49	50
WK15	56	55	56	53
WK36	58	53	55	55

Figure S1 Longitudinal cohort of infants born to mother living with HIV (HIV-exposed-uninfected infants) and infants born to mother without HIV (HIV-unexposed uninfected). Infants received childhood vaccines including acellular pertussis vaccine at weeks 6, 10 & 14, and rotavirus vaccine at weeks 6 & 14. Blood samples was collected from cord blood (CB) and infants at birth (< 12hrs postpartum), and weeks 4, 15 and 36 for isolation of peripheral blood mononuclear cells (PBMC) and plasma. PBMC samples were fractioned for immunophenotyping of NK cells and T cells using mass cytometry and for T cell receptor RNA-sequencing of sorted naïve and memory CD4+ and CD8+ T cells. Plasma samples were used to measure anti-pertussis IgG levels at birth (BTH) and weeks (WK) 4, 15 and 36 and anti-IgA titre and neutralization at week 36. Figure partially generated using Biorender.

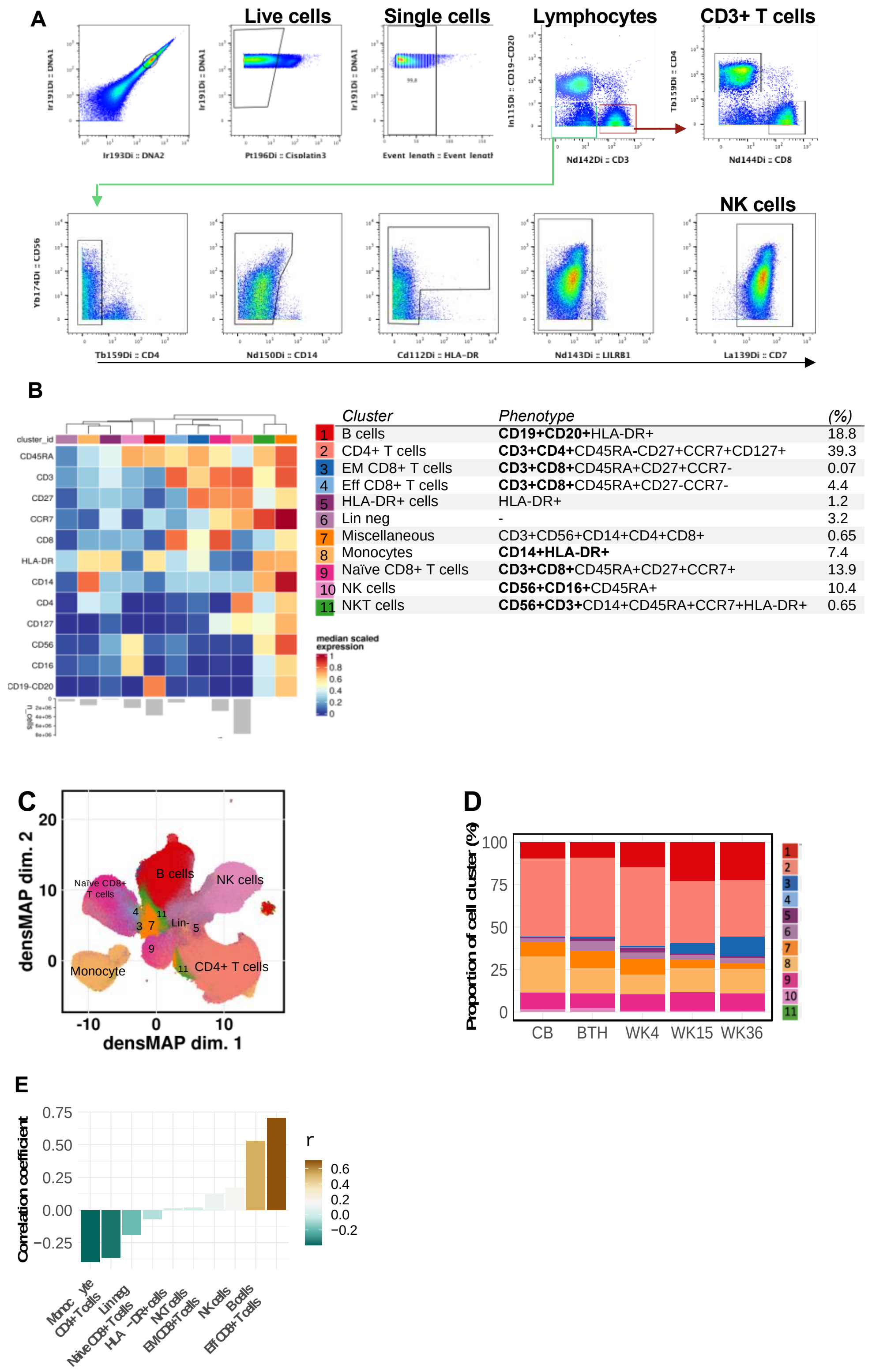

Cluster		Phenotype	(%)
1	B cells	**CD19+CD20+**HLA-DR+	18.8
2	CD4+ T cells	**CD3+CD4+**CD45RA-CD27+CCR7+CD127+	39.3
3	EM CD8+ T cells	**CD3+CD8+**CD45RA+CD27+CCR7-	0.07
4	Eff CD8+ T cells	**CD3+CD8+**CD45RA+CD27-CCR7-	4.4
5	HLA-DR+ cells	HLA-DR+	1.2
6	Lin neg	-	3.2
7	Miscellaneous	CD3+CD56+CD14+CD4+CD8+	0.65
8	Monocytes	**CD14+HLA-DR+**	7.4
9	Naïve CD8+ T cells	**CD3+CD8+**CD45RA+CD27+CCR7+	13.9
10	NK cells	**CD56+CD16+**CD45RA+	10.4
11	NKT cells	**CD56+CD3+**CD14+CD45RA+CCR7+HLA-DR+	0.65

Figure S2 Immunophenotype of lineage immune cell clusters. **A)** Gating strategy used to determine live singlet cells of CD4+ and CD8+ T cells and NK cells subsets. **B)** Heatmap showing scaled marker expression for FlowSOM immune lineage clusters derived from live singlet cells. **C)** Uniform manifold approximation and proximation (UMAP) with density preservation showing dimensional reduction of ineage immune cell clusters across all time points. **D)** Relative abundance of each immune cell cluster dentified in infant samples collected in cord blood (CB), infant peripheral blood at birth (BTH), and weeks (WK) 4, 15, and 36. **E)** Spearman's rank correlation between the proportion of immune cell clusters and infant age from birth until week 36.

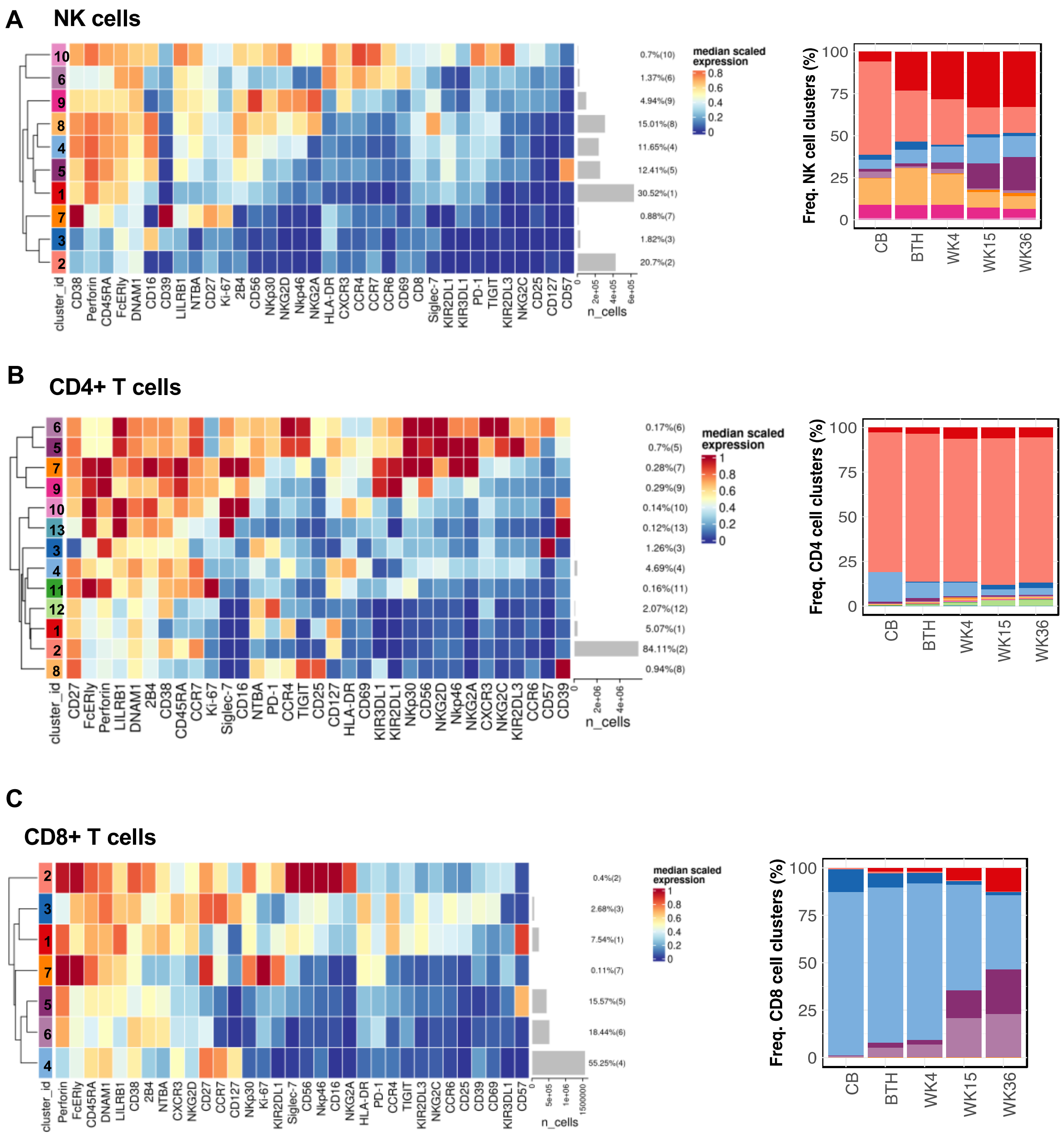

Figure S3 Immunophenotype of NK cell, and CD4+ and CD8+ T cell clusters. **A-C)** Heatmap showing re-scaled marker expression for cell clusters derived from NK cells CD4+ and CD8+ T cells and associated relative abundance of each cell cluster measured in cord blood (CB) and infant peripheral blood at birth (BTH), and weeks (WK) 4, 15 and 36.

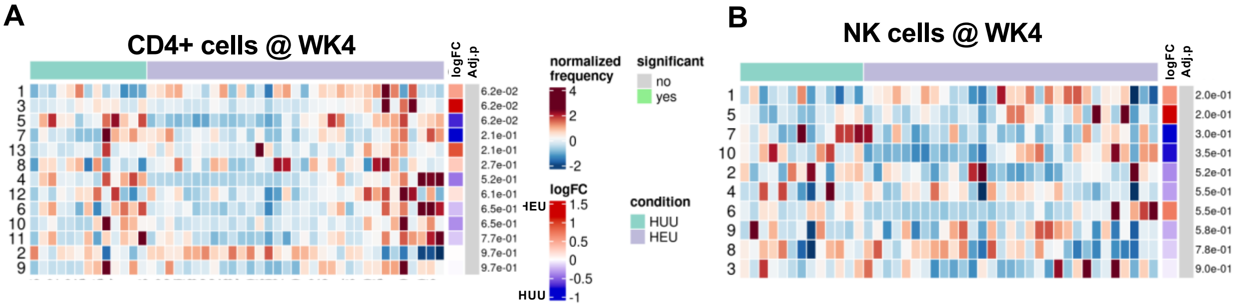

Figure S4 A&B) GLMM comparing the abundances of NK cell and CD4+ T cell clusters between iHEU and iHUU at weeks 4 respectively. Positive log Fold change (FC) signified cell cluster frequencies were higher in iHEU compared to iHUU. P-value adjusted for multiple comparison using FDR.

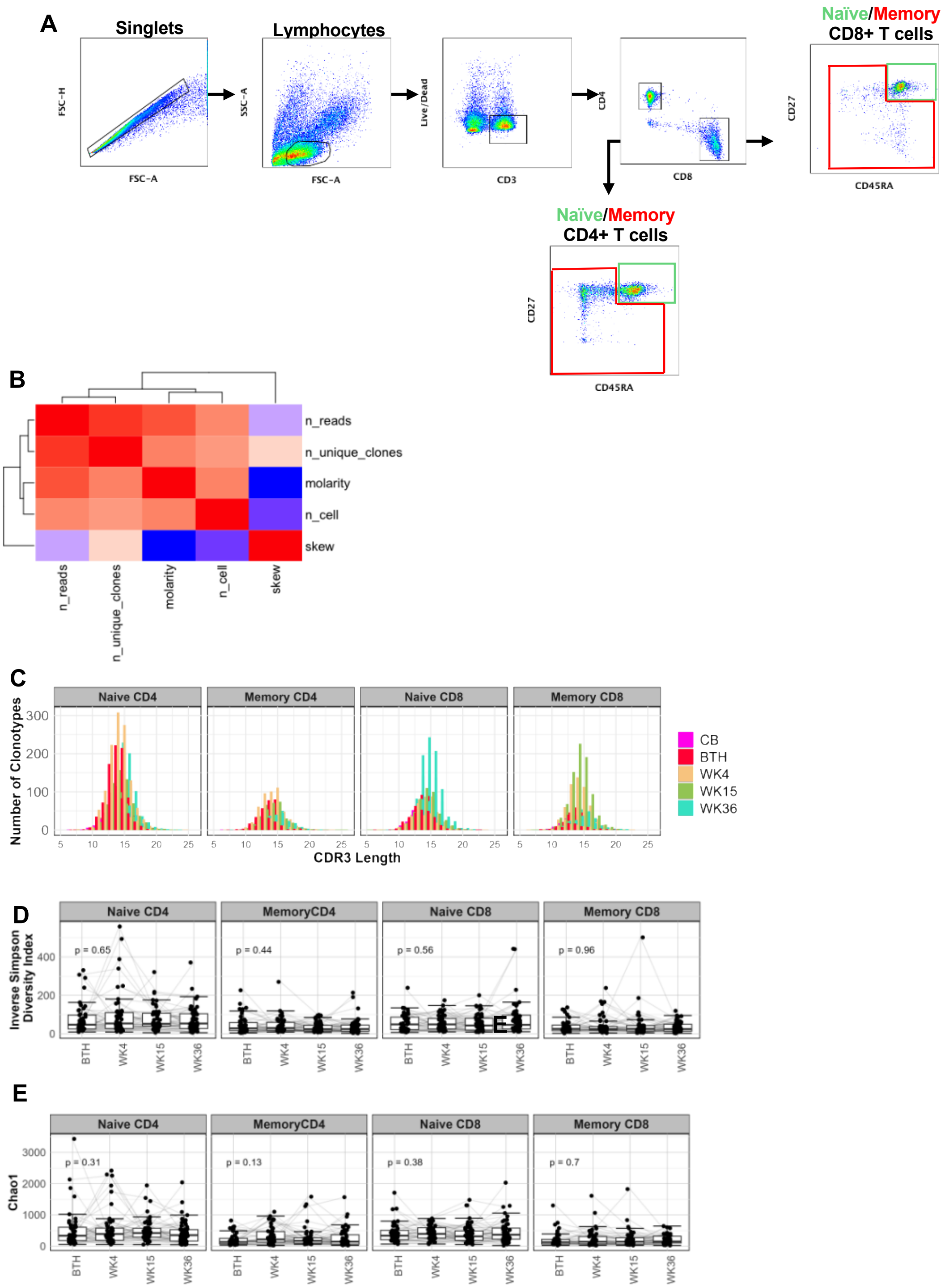

Figure S5 Naïve and memory CD4^{+} and CD8^{+} T cell receptor (TCR) repertoire in HIV-exposed uninfected infants (iHEU) and HIV-unexposed uninfected infants (iHUU). **A)** Flowplots showing gating strategy for sorting naïve and memory CD4+ and CD8+ T cells in infants' peripheral blood mononuclear cells prior TCR RNA sequencing. **B)** Heatmap showing correlation matrix of TCR quality control parameters. **C)** CDR3 lengths distribution between iHUU and iHEU. **D)** Longitudinal changes in TCR diversity scores measured by Inverse Simpson index. **E)** Longitudinal changes in TCR richness scores measured by Choa1 index. All boxplots used the standard Tukey's representation with the central line depicting the median, the upper and lower lines represent the 75th and 25th percentile respectively, and the whiskers mark the boundary 1.5 times of the 75th and 25th percentile. P-value determined using Kruskal-Wallis test.

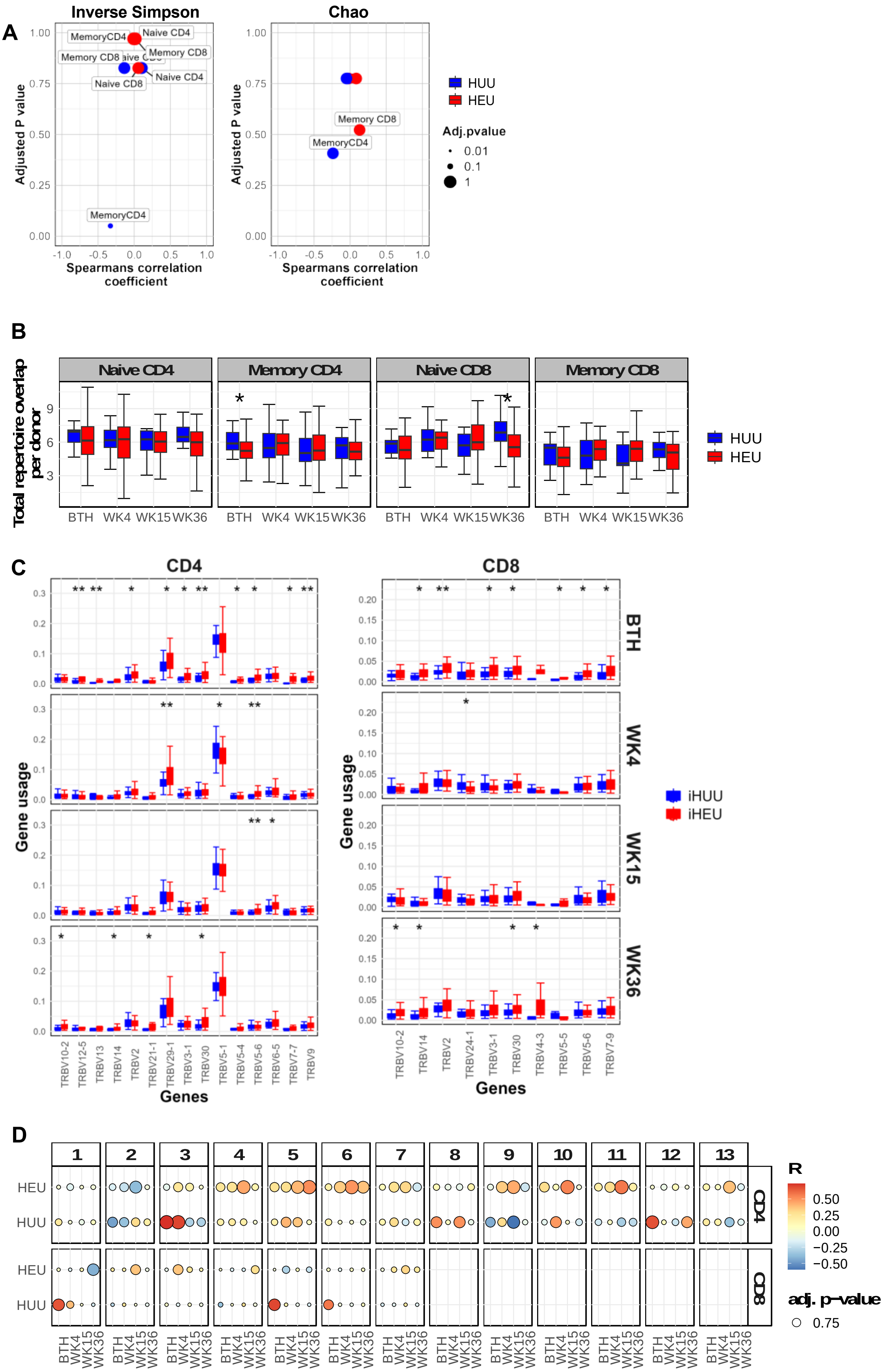
A
Inverse Simpson
Chao
Adjusted P value
Spearmans correlation coefficient
MemoryCD4
Naive CD4
Memory CD8
Naive CD8
HUU
HEU
Adj.pvalue
0.01
0.1
1
B
Naive CD4
Memory CD4
Naive CD8
Memory CD8
Total repertoire overlap per donor
BTH
WK4
WK15
WK36
C
CD4
CD8
Gene usage
Genes
iHUU
iHEU
TRBV10-2
TRBV12-5
TRBV13
TRBV14
TRBV2
TRBV21-1
TRBV29-1
TRBV3-1
TRBV30
TRBV5-1
TRBV5-4
TRBV5-6
TRBV6-5
TRBV7-7
TRBV9
TRBV24-1
TRBV4-3
TRBV5-5
TRBV7-9
D
R
adj. p-value
0.75

Figure S6 T cell receptor (TCR) structural repertoire and gene usage in HIV-exposed uninfected infants (iHEU) and HIV-unexposed uninfected infants (iHUU). **A)** Spearma's correlation between infant age in weeks and Inverse Simpson diversity scores and Chao richness scores of naïve and memory CD4+ and CD8+ T cells. **B)** Comparing TCR repertoire structural overlap as measured using Jaccard-indeces between iHEU and iHUU at BTH, and weeks (WK) 4, 15 and 36. **C)** Comparing TCR Vβ gene usages between I HEU and iHUU in CD4+ and CD8+ T cells measured at BTH and WK 4, 15 and 36. D) Spearman's rank correlation between memory CD4+ and CD8+ T cell Inverse Simpson scores and frequencies of CD4+ and CD8+ T cell clusters at week 15 and 36. **D)** Spearman's rank correlation between memory CD4+ and CD8+ T cell Inverse Simpson scores and frequencies of CD4+ and CD8+ T cell clusters respectively. *adjusted $p<0.05$; ** adjusted $p<0.01$, FDR used to correct for multiple comparisons.

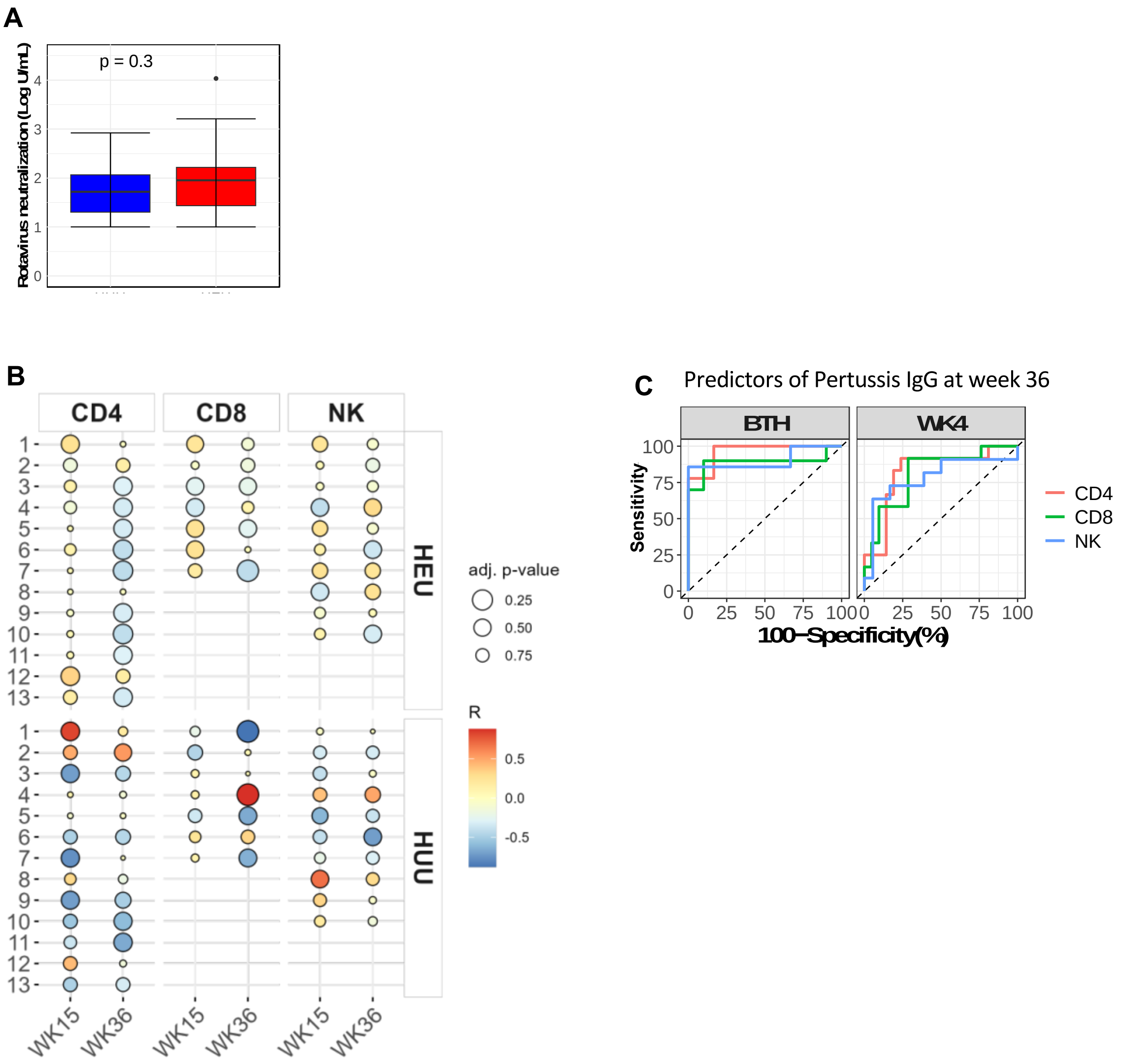

Figure S7 Vaccine induced antibody responses in HIV-exposed uninfected infants (iHEU) and HIV-unexposed uninfected (iHUU). **A**) Boxplots comparing anti-rotavirus IgA neutralization titres between iHEU and iHUU at week 36. Boxplot central line depicts the median, the upper and lower lines represents the 75th and 25th percentile respectively, and the whiskers mark the boundry 1.5 times the 75th and 25th percentile boundary, (Two-tailed Wilcoxon) **B)** Spearman's correlation between abundances of FlowSOM clusters for NK cells, and CD4+ and CD8+ T cells and anti-pertussis IgG titres measured at week 15 and 36 in iHEU and iHUU. P-value adjusted for multiple comparisons using FDR. **C)** Summary of ROC analysis using the latent variable axis-1 derived from partial least square discriminate analysis (PLS-DA) of NK, CD4 and CD8 T cell clusters determined to be best predictors at birth and week 4 of pertussis antibody responses at weeks 36.

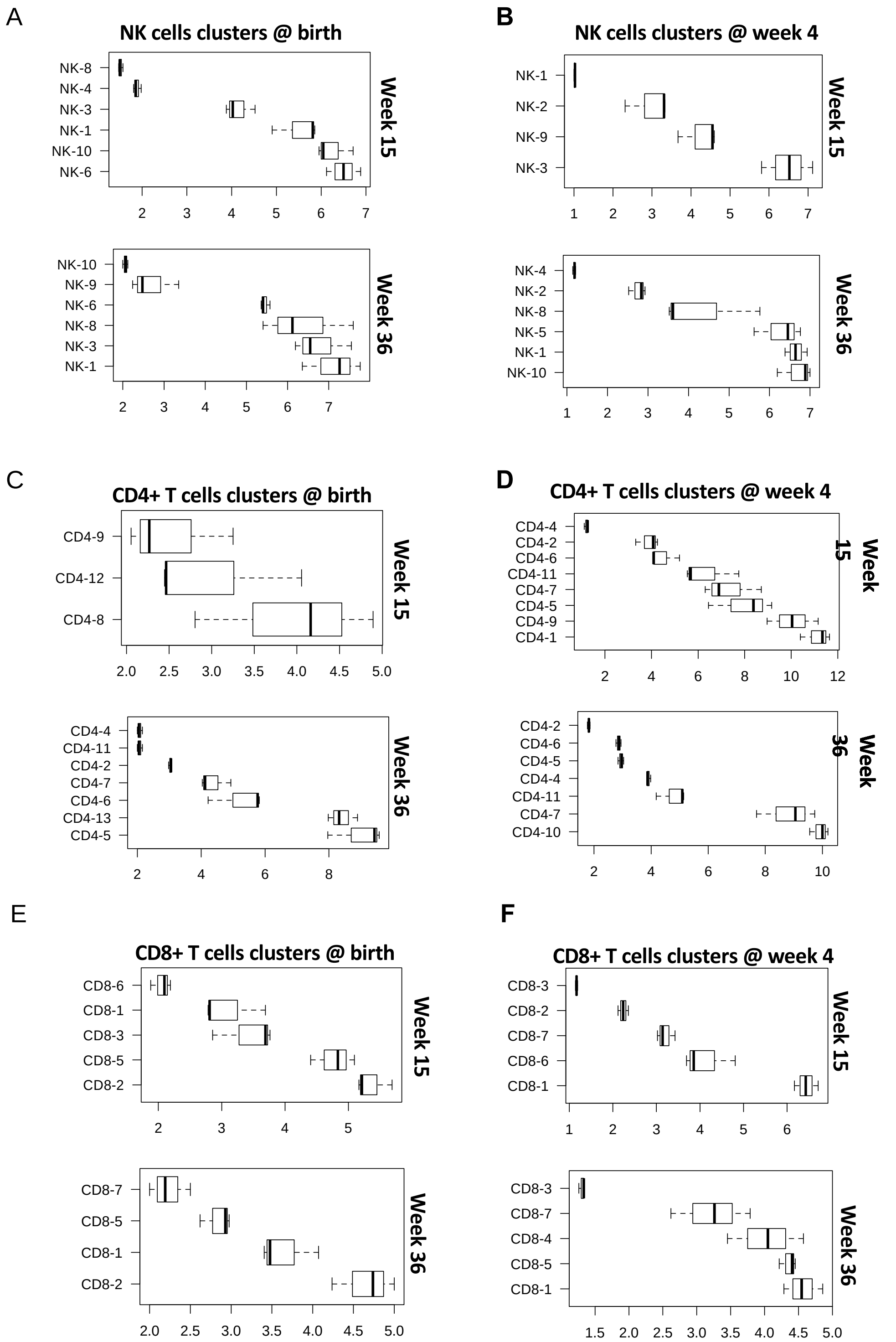

Figure S8 Immune cell clusters predictive of pertussis specific antibody responses post-vaccination. Multivariable regression using partial least squares with discriminate analysis (PLS-DA) and recursive variable elimination within repeated double cross-validation for selection of the minimum number of cell clusters with low misclassification error for predicting pertussis specific IgG responses at week 15 and 36. **A & B)** NK cell clusters predictors at birth and week 4 respectively. **C & D)** CD4+ T cell predictors at birth and week 4 respectively. **E & F)** CD8+ T cell predictors at birth and week 4 respectively.

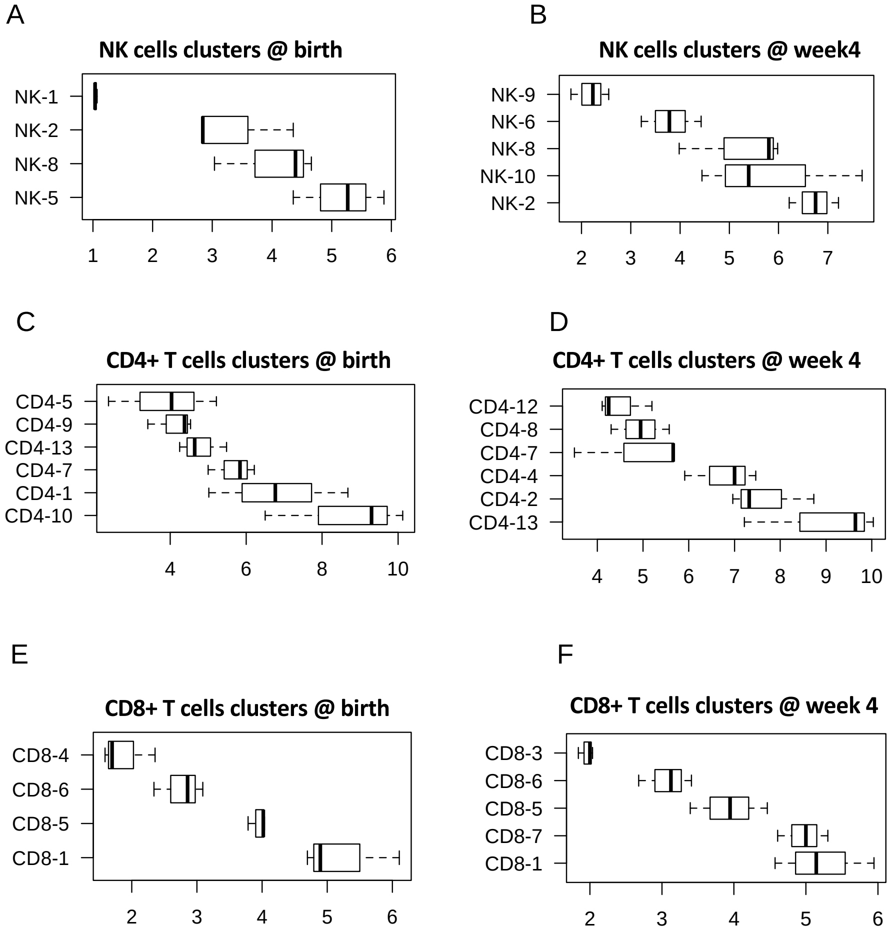

Figure S9 Immune cell clusters predictive of rotavirus specific antibody responses post-vaccination. Multivariable regression using partial least squares with discriminate analysis (PLS-DA) and recursive variable elimination within repeated double cross-validation for selection of the minimum number of cell clusters with low misclassification error for predicting rotavirus specific IgG responses at week 36. **A & B)** NK cell clusters predictors at birth and week 4 respectively. **C & D)** CD4+ T cell predictors at birth and week 4 respectively. **E & F)** CD8+ T cell predictors at birth and week 4, respectively.
