# Supplementary material for: Premature skewing of T cell receptor clonality and delayed memory expansion in HIV-exposed infants
Source: Nat Commun. 2024 May 14;15:4080. doi: 10.1038/s41467-024-47955-5 (PMC11093981; doi:10.1038/s41467-024-47955-5)
Supplement: Supplementary file 3 — Description of Additional Supplementary Files [file 41467_2024_47955_MOESM3_ESM.pdf]

Description of Additional Supplementary Files

File Name: Data S1
Description: Summary of T cell receptor sequencing quality control parameters used for naïve and memory T cells measured at birth, and weeks 4, 15 and 36. *p-value<0.05; two-tailed Wilcoxon test, adjusted for multiple comparisons using FDR.
