# Supplementary material for: Premature skewing of T cell receptor clonality and delayed memory expansion in HIV-exposed infants
Source: Nat Commun. 2024 May 14;15:4080. doi: 10.1038/s41467-024-47955-5 (PMC11093981; doi:10.1038/s41467-024-47955-5)
Supplement: Supplementary file 5 — Reporting Summary [file 41467_2024_47955_MOESM5_ESM.pdf]

nature portfolio

Corresponding author(s): Clive Gray; Catherine Blish and Heather Jaspan

Last updated by author(s): Mar 25, 2024

Reporting Summary

Nature Portfolio wishes to improve the reproducibility of the work that we publish. This form provides structure for consistency and transparency in reporting. For further information on Nature Portfolio policies, see our Editorial Policies and the Editorial Policy Checklist.

Statistics

For all statistical analyses, confirm that the following items are present in the figure legend, table legend, main text, or Methods section.

n/a	Confirmed	
☐	☒	The exact sample size (*n*) for each experimental group/condition, given as a discrete number and unit of measurement
☐	☒	A statement on whether measurements were taken from distinct samples or whether the same sample was measured repeatedly
☐	☒	The statistical test(s) used AND whether they are one- or two-sided<br>*Only common tests should be described solely by name; describe more complex techniques in the Methods section.*
☐	☒	A description of all covariates tested
☐	☒	A description of any assumptions or corrections, such as tests of normality and adjustment for multiple comparisons
☐	☒	A full description of the statistical parameters including central tendency (e.g. means) or other basic estimates (e.g. regression coefficient) AND variation (e.g. standard deviation) or associated estimates of uncertainty (e.g. confidence intervals)
☐	☒	For null hypothesis testing, the test statistic (e.g. *F*, *t*, *r*) with confidence intervals, effect sizes, degrees of freedom and *P* value noted<br>*Give P values as exact values whenever suitable.*
☒	☐	For Bayesian analysis, information on the choice of priors and Markov chain Monte Carlo settings
☐	☒	For hierarchical and complex designs, identification of the appropriate level for tests and full reporting of outcomes
☐	☒	Estimates of effect sizes (e.g. Cohen's *d*, Pearson's *r*), indicating how they were calculated

Our web collection on statistics for biologists contains articles on many of the points above.

Software and code

Policy information about availability of computer code

Data collection

Mass cytometry data was generated using CyTOFII instrument from DVS Sciences.
Cells were sorted using BD FACSAria II.
FCS files generated from mass cytometry and FACS eperiments were a gated using FlowJo version 10.5.3.
T cell receptor (TCR) sequencing was performed using Illumina NovaSeq SP. ELISA optical density absorbence was measured using Molecular Devices SpectraMax plus plate reader.

Data analysis

Data was analyzed using R open software. No custom code was developed and all packages used are described in the method section.
Mass cytometry analysis: R packages were used in chronological order: Premessa (data normalisation), scDblFinder t(doublet cell removal - computeDoubletDenisity function), stats (determine Multidimensional scaling ordinates - cmdscale function), CATALYST (unsupervised cell clustering - FlowSOM and ConsensusClusterPlus functions, and generalised linear mixed model - diffcyt function), denvis (compute UMAPS with density preservation - densMAP function), and
TCR data analysis: REpertoire Sequencing TOolkit (pRESTO) was used to for processing FASTQC files and MiXCR software used to read mapping. The immunarch R package was immune repertoire analysis including inverse Simposon Index, Chao richness score, CDR3 length and TCR V-beta gene usage. Antigen specificities were predicted using GLIPH2 algorithm (grouping of lymphocyte interactions by paratope hotspots).
Antibody responses were analyzed using R packages including corrplot (Spearman's correlation - correlation function), rstatix (pairwaise comparison and multiple comparison correction), MUVR (unbiased selection of predictive variables) and mixOmics (partial least square with discriminant analysis).

For manuscripts utilizing custom algorithms or software that are central to the research but not yet described in published literature, software must be made available to editors and reviewers. We strongly encourage code deposition in a community repository (e.g. GitHub). See the Nature Portfolio guidelines for submitting code & software for further information.

Data

Policy information about availability of data

All manuscripts must include a data availability statement. This statement should provide the following information, where applicable:

- Accession codes, unique identifiers, or web links for publicly available datasets
- A description of any restrictions on data availability
- For clinical datasets or third party data, please ensure that the statement adheres to our policy

CyTOF data that was used for analysis infant immune trajectories and FACS files generated when sorting for naive and memory CD4+ and CD8+ T cells were uploaded to ImmPort repository (SDY2463). FastQC files generated from TCR RNA sequencing data are available at Gene Expression Omnibus (GSE256410) Additional data files used in this study were uploaded to Figshare repository.

Research involving human participants, their data, or biological material

Policy information about studies with human participants or human data. See also policy information about sex, gender (identity/presentation), and sexual orientation and race, ethnicity and racism.

Reporting on sex and gender	This study was limited to analysis infant samples and as such only biological sex of the infants has been reported. Sex was not part of the study inclusion and/or exclusion criteria.
Reporting on race, ethnicity, or other socially relevant groupings	All of the enrolled study participant were Black South Africans since the clinical recruitment site is situated in Khayelitsh, Cape Town, South Africa.
Population characteristics	The study included 46 infants from the INFANT cohort, which consisted of infants born to mothers living with HIV, although the infants remained HIV free and were denoted as HIV-exposed uninfected infants (iHEU, n=40) and compared to those infants born to mother without HIV; referred to as HIV-unexposed uninfected infants (iHUU, n=16). Infant population characteristics were similar between the iHEU and iHUU at delivery including median gestational age (39 weeks, IQR:36-42 vs 39 weeks, IQR:36-41, respectively), median birth weight (3.1 kg; IQR: 2.9-3.4 vs 3.2kg; IQR: 2.8-3.5 kg, respectively) and sex distribution (Females = 0.5 vs 0.35, respectively). Other infant variables were also consistent between the two groups of infants such as median duration of breast feeding (iHEU=52 weeks vs iHUU =44.3 weeks) proportion of infants completed rotavirus vaccination (iHEU=0.88 vs iHUU=0.81) and pertussis vaccination (iHEU=0.83 vs iHUU=0.75) compared in the study. The median age for mothers of iHEU were significantly older at delivery compared to those of iHUU (30.5 years vs 23.5 years, p=0.02). Descriptive population characteristics are presented in supplementary table S1 of the manuscript.
Recruitment	Pregnant women attending antenatal clinical visits at Site B MOU clinic in Khayelitsha, Cape Town (2012-2018) were recruited to enroll themselves and their infants to the study. This included woman who knew their HIV status and/or willing to undergo HIV testing. The mothers provided signed informed consent to enroll themselves and their infants to the study. Infants who tested positive for HIV DNA PCR were excluded from study. The study also excluded infants who were delivered < 37 weeks gestation and having birth weight < 2.5 kg. Infants were enrolled in the study at birth and were followed up at weeks 4, 15 and 36. The study participants received ZAR150 at each study visit as compensation of transport and working hours lost.
Ethics oversight	Human Research Ethics Committee of University of Cape Town approved the study (HREC#285/2012)

Note that full information on the approval of the study protocol must also be provided in the manuscript.

Field-specific reporting

Please select the one below that is the best fit for your research. If you are not sure, read the appropriate sections before making your selection.

☒ Life sciences ☐ Behavioural & social sciences ☐ Ecological, evolutionary & environmental sciences

For a reference copy of the document with all sections, see nature.com/documents/nr-reporting-summary-flat.pdf

Life sciences study design

All studies must disclose on these points even when the disclosure is negative.

Sample size	Our previous data comparing Treg/Th17 ontogeny in a similar cohort was used as a guideline for the sample size determination. However, since we aimed to analyze longitudinal immune changes in infants we were limited by identifying infants with samples collected at all study visits (birth, weeks 4, 15 and 36).
Data exclusions	We excluded CyTOF FCS files with fewer than 1000 cells/events. The threshold was not pre-established, but was implemented due to low cell counts obtained from select infants. TCR-seq results were the unique clones were higher than the total cell count sequences were also excluded.
Replication	CyTOF data - Of the 236 infant PBMC samples analysed, 27 had >2 vials of stored PBMC and were included as replicates, and 2 adult

Replication	controls were included to validate the data.<br>ELISA experiments - All performed in duplicates. CV values included in shared data.
Randomization	Study used archived samples collected from a previous study. We prioritized infants with PBMC collected at multiple time points for our longitudinal study
Blinding	All samples collected in the study are allocated a blinding ID and only after data acquisition was the investigators unblinded.

Reporting for specific materials, systems and methods

We require information from authors about some types of materials, experimental systems and methods used in many studies. Here, indicate whether each material, system or method listed is relevant to your study. If you are not sure if a list item applies to your research, read the appropriate section before selecting a response.

Materials & experimental systems

n/a	Involved in the study
☐	☒ Antibodies
☐	☒ Eukaryotic cell lines
☒	☐ Palaeontology and archaeology
☒	☐ Animals and other organisms
☐	☒ Clinical data
☒	☐ Dual use research of concern
☒	☐ Plants

Methods

n/a	Involved in the study
☒	☐ ChIP-seq
☐	☒ Flow cytometry
☒	☐ MRI-based neuroimaging

Antibodies

Antibodies used	Antibodies for cell labeling are listed in TableS2. These are commercial antibodies obtained from Biolegend, BD BioSciences, R&D systems, and etc.<br>CyTOF analysis the following antibodies were used:<br>anti-CD19 In115Di (clone: HIB19, cat#302247, Biolegend); anti-CD20 In115Di (clone: 2H7, cat#302343, Biolegend); anti-CD14 Nd150Di (clone: M5E2, cat#301843, Biolegend); anti-CD3 Nd142Di (clone: UCHT1, cat#300443, Biolegend); anti-CD4 Tb159Di (clone: OKT4, cat#317402, Biolegend); anti-CD8 Nd144Di (clone: SK1, cat#344727, Biolegend); anti-CD45RA Nd148Di (clone: HI100, cat#304143, Biolegend); anti-KIR2DL1 Sm149Di (clone: 143211, cat#328302, R&D Systems); anti-CD57 Eu151Di (clone: HCD57, cat#359602, Biolegend); anti-Siglec7 Eu153Di (clone: S7.7, cat#339202, Biolegend); anti-PD1 Sm154Di (clone: EH12.2H7, cat#329941, Biolegend); anti-NKp46 Gd155Di (clone: 900, cat#331902, Biolegend); anti-NKG2D Gd156Di (clone: 1D11, cat#320802, Biolegend); anti-NKG2C Gd157Di (clone: 134591, cat# MAB138, R&D System); anti-2B4 Gd158Di (clone: C1.7, cat#329502, Biolegend); anti-CXCR3 Gd160Di (clone: G025H7, cat#353733, Biolegend); NKp30 Dy161Di (clone: P30.15, cat#325202, Biolegend); anti-CD39 Dy162Di (clone: A1, cat#328221, Biolegend); KIR3DL1 Dy163Di (clone: DX-9, cat#555964, BD biosciences); anti-TIGIT Dy164Di (clone: 741182, cat# MAB7898, R&D System); anti-CD16 Ho165Di (clone: 3G8, cat#302051, Biolegend); anti-CD69 Er166Di (clone: FN50, cat#310939, Biolegend); anti-CD127 Er167Di (clone: A019D5, cat#351337, Biolegend); anti-CCR7 Er168Di (clone: G043H7, cat#353237, Biolegend); anti-NKG2A Tm169Di (clone:Z199, cat#3169013B, Fluidigm); anti-KIR2DL3 Er170Di (clone: 180701, cat# MAB2014, R&D System); anti-CCR4 Yb171Di (clone: 1G1, clone:, cat#551121, BD Biosciences); anti-NTBA Yb172Di (clone: NT-7, cat#317202, Biolegend); anti-CCR6 Yb173Di (clone: G034E3, cat#353427, Biolegend); anti-CD56 Yb174Di (clone: NCAM16.2, cat#559043, BD Biosciences); anti-CD25 Lu175Di (clone: M-A251, cat#356102, Biolegend); anti-CD38 Yb176Di (clone: HIT2, cat#303535, Biolegend); anti-CD7 La139Di (clone: CD7-6B7, cat#343111, Biolegend); anti-DNAM1 Pr141Di (clone: DX11, cat#559787, BD Biosciences); anti-LILRB1 Nd143Di (clone: 292319, cat# MAB20172, R&D System); anti-CD27 Nd146Di (clone: O323, cat#302839, Biolegend); anti-HLA-DR Cd112Di (clone: Tu36, cat#361602, Biolegend); anti-Ki67 Sm152Di (clone: Ki-67, cat#350523, Biolegend); anti-Perforin Sm147Di (clone: B-D48, cat# ab47225, Abcam); anti-FcERIy Nd145Di (clone: Polyclonal, cat#06-727,Millipore).<br><br>FACS sorting the following antibodies were used:<br>anti-CD3 FITC (clone: OKT3, cat#317306, Biolegend); anti-CD4 Alexa Flour 700 (clone: SK3, cat#344622 Biolegend); anti-CD8 BV711 (clone: RPA-T8, cat#301043 Biolegend); anti-CD45RA PE-Texas red (clone: MEM-56, cat# MHCD45RA17 Invitrogen); anti-CD27 PE-Cy5 (clone: G043H7, cat#302858 Biolegend); anti-CCR7 PE-Cy7 (clone: O323, cat#353225 Biolegend)<br><br>Antibody quantification<br>Rabbit Anti-Rotavirus IgA (cat#ab93860, Abcam); biotyinylated goat anti-human IgA (cat#09-065-011, Jackson Laboratories)
Validation	Supplier validation and internal antibody screening.

Eukaryotic cell lines

Policy information about cell lines and Sex and Gender in Research

Cell line source(s)	NIH
Authentication	None

Mycoplasma contamination	Cell lines tested negative for mycoplasma contamination.
Commonly misidentified lines (See ICLAC register)	MA104 (monkey kidney cells) determined to be contaminated by human HeLa cells

Clinical data

Policy information about clinical studies

All manuscripts should comply with the ICMJE guidelines for publication of clinical research and a completed CONSORT checklist must be included with all submissions.

Clinical trial registration	*Provide the trial registration number from ClinicalTrials.gov or an equivalent agency.*
Study protocol	*Note where the full trial protocol can be accessed OR if not available, explain why.*
Data collection	*Describe the settings and locales of data collection, noting the time periods of recruitment and data collection.*
Outcomes	*Describe how you pre-defined primary and secondary outcome measures and how you assessed these measures.*

Plants

Seed stocks	*Report on the source of all seed stocks or other plant material used. If applicable, state the seed stock centre and catalogue number. If plant specimens were collected from the field, describe the collection location, date and sampling procedures.*
Novel plant genotypes	*Describe the methods by which all novel plant genotypes were produced. This includes those generated by transgenic approaches, gene editing, chemical/radiation-based mutagenesis and hybridization. For transgenic lines, describe the transformation method, the number of independent lines analyzed and the generation upon which experiments were performed. For gene-edited lines, describe the editor used, the endogenous sequence targeted for editing, the targeting guide RNA sequence (if applicable) and how the editor was applied.*
Authentication	*Describe any authentication procedures for each seed stock used or novel genotype generated. Describe any experiments used to assess the effect of a mutation and, where applicable, how potential secondary effects (e.g. second site T-DNA insertions, mosiacism, off-target gene editing) were examined.*

Flow Cytometry

Plots

Confirm that:

☒ The axis labels state the marker and fluorochrome used (e.g. CD4-FITC).

☒ The axis scales are clearly visible. Include numbers along axes only for bottom left plot of group (a 'group' is an analysis of identical markers).

☒ All plots are contour plots with outliers or pseudocolor plots.

☒ A numerical value for number of cells or percentage (with statistics) is provided.

Methodology

Sample preparation	Infant blood samples were used to isolate peripheral blood mononuclear cells (PBMC) were using Ficoll gradient centrifugation. PBMC were stored in Fecal calf serum supplemented with 10% DMSO.
Instrument	CyTOF data was generated using CyTOFII from DVS Sciences and FACS analysis was performed using BD FACS-Aria Fusion (BD)
Software	FlowJo version 10.5.3 and R openware was used for data analysis
Cell population abundance	We obtained >95% post sort purity.
Gating strategy	CyTOF - cells were gated for Live CD3+ T cells and NK cells (CD14-CD19-CD3-CD4-). Populations of CD4+ and CD8+ T cells and NK cells were identified using unsupervised cell clustering from the CATALYST package in R.

☒ Tick this box to confirm that a figure exemplifying the gating strategy is provided in the Supplementary Information.
